# Stability and Hopf Bifurcation in a Delayed HIV Infection Model with General Incidence Rate and Immune Impairment

**DOI:** 10.1155/2015/206205

**Published:** 2015-08-04

**Authors:** Fuxiang Li, Wanbiao Ma, Zhichao Jiang, Dan Li

**Affiliations:** ^1^Department of Applied Mathematics, School of Mathematics and Physics, University of Science and Technology Beijing, Beijing 100083, China; ^2^Fundamental Science Department, North China Institute of Aerospace Engineering, Langfang, Hebei 065000, China

## Abstract

We investigate the dynamical behavior of a delayed HIV infection model with general incidence rate and immune impairment. We derive two threshold parameters, the basic reproduction number *R*_0_ and the immune response reproduction number *R*_1_. By using Lyapunov functional and LaSalle invariance principle, we prove the global stability of the infection-free equilibrium and the infected equilibrium without immunity. Furthermore, the existence of Hopf bifurcations at the infected equilibrium with CTL response is also studied. By theoretical analysis and numerical simulations, the effect of the immune impairment rate on the stability of the infected equilibrium with CTL response has been studied.

## 1. Introduction

In recent years, mathematical models have been proved to be valuable in understanding the dynamics of viral infection (see, e.g., [1–8]). In most virus infections, cytotoxic T lymphocyte (CTL) cells play a significant role in antiviral defense by attacking virus-infected cells. In order to study the role of the population dynamics of the viral infection with CTL response, Nowak and Bangham et al. proposed a basic viral infection model describing the interactions between a replicating virus population and a specific antiviral CTL response, which takes into account four populations: uninfected cells, actively infected cells, free virus, and CTL cells (see, e.g., [1–4, 9, 10]). Now, the population dynamics of viral infection with CTL response has been paid much attention and many properties have been investigated (see, e.g., [11–16]).

Furthermore, the state of latent infection cannot be ignored in many biological models. The infected cells are separated into two distinct compartments, latently infected and actively infected. These latently infected cells do not produce virus and can evade from viral cytopathic effects and host immune mechanisms (see, e.g., [17–20]). Recently, the following model with latent infection and CTL response has been proposed (see, e.g., [11]):

(1) x˙t=λ−βxtvt−μ1xt,u˙t=βxtvt−σ+μ2ut,y˙t=σut−pytzt−μ3yt,v˙t=kyt−μ4vt,z˙t=qytzt−μ5zt,

where *x*(*t*), *u*(*t*), *y*(*t*), *v*(*t*), and *z*(*t*) represent the numbers of uninfected cells, latently infected cells, actively infected cells, free virus, and CTLs at time *t*, respectively. Uninfected cells are produced at the rate *λ*, die at the rate *μ*_1_, and become infected at the rate *β*. The constant *σ* is the rate of latently infected cells translating to actively infected cells and *μ*_3_ is the death rate of actively infected cells. The constant *μ*_2_ represents the death rate of latently infected cells. The constant *p* is the rate of CTL-mediated lysis and *q* is the rate of CTL proliferation. The constant *k* is the rate of production of virus by infected cells and *μ*_4_ is the clearance rate of free virus. The removal rate of CTLs is *μ*_5_.

However, in plenty of previous papers, many models are constructed under the assumption that the presence of antigen can stimulate immunity and ignore the immune impairment (see, e.g., [8, 11, 16, 17]). In fact, some pathogens can also suppress immune response or even destroy immunity especially when the load of pathogens is too high such as HIV, HBV (see, e.g., [15, 21–25]). Regoes et al. consider an ordinary differential equation (ODE) model with an immune impairment term *myz* (see, e.g., [12, 26, 27]), where *m* denotes the immune impairment rate. Time delay should be considered in models for CTL response. It is shown that time delay plays an important role to the dynamic properties in models for CTL response (see, e.g., [1, 5, 6, 8, 15]). In fact, antigenic stimulation generating CTLs may need a period of time *t*; that is, the CTL response at time *t* may depend on the numbers of CTLs and infected cells at time *t* − *τ*, for a time lag *τ* > 0 (see, e.g., [1, 5, 13]).

Motivated by the above works, in this paper, we will study a delay differential equation (DDE) model of HIV infection with immune impairment and delayed CTL response. Furthermore, we know that the actual incidence rate is probably not linear over the entire range of *x* and *v*. Based on the works mentioned above (see, e.g., [21, 28–31]), we propose the following system with general incidence function:

(2) x˙t=λ−fxt,vtvt−μ1xt,u˙t=fxt,vtvt−σ+μ2ut,y˙t=σut−pytzt−μ3yt,v˙t=kyt−μ4vt,z˙t=qyt−τzt−τ−μ5zt−mytzt,

where the state variables *x*(*t*), *u*(*t*), *y*(*t*), *v*(*t*), and *z*(*t*) and the parameters *λ*, *σ*, *p*, *k*, *q*, *μ*_1_, *μ*_2_, *μ*_3_, *μ*_4_, and *μ*_5_ have the same biological meaning as in system (1). *m* is the immune impairment rate. Suppose all the parameters are nonnegative. We assume the incidence rate is the general incidence function *f*(*x*, *v*)*v*, where *f* ∈ *C* ^1^([0, +*∞*]×[0, +*∞*], *R*) satisfies the following hypotheses:

(H1) *f*(*x*, *v*)*v* ≥ 0, for all *x* ≥ 0 and *v* ≥ 0; *f*(*x*, *v*) = 0 if and only if *x* = 0;
(H2) ∂*f*(*x*, *v*)/∂*x* > 0, for all *x* ≥ 0 and *v* ≥ 0;
(H3) ∂*f*(*x*, *v*)/∂*v* ≤ 0, for all *x* ≥ 0 and *v* ≥ 0;
(H4) ∂(*f*(*x*, *v*)*v*)/∂*v* > 0, for all *x* > 0 and *v* ≥ 0.

Clearly, the hypotheses can be satisfied by different types of the incidence rate including the mass action, the Holling type II function, the saturation incidence, Beddington-DeAngelis incidence function, Crowley-Martin incidence function, and the more generalized incidence functions (see, e.g., [4, 6, 17, 32, 33]). Further, in order to study the global stability of the equilibria of system (2) by the method of Lyapunov functionals, we assume the following hypotheses hold (see, e.g., [28]):

(H5) *x* − *x*_0_ − ∫_*x*_0__^*x*^(*f*(*x*_0_, 0)/*f*(*s*, 0))*ds* → +*∞*, as *x* → +*∞* or *x* → 0^+^;
(H6) *x* − *x*_1_ − ∫_*x*_1__^*x*^(*f*(*x*_1_, *v*_1_)/*f*(*s*, *v*_1_))*ds* → +*∞*, as *x* → +*∞* or *x* → 0^+^;
(H7) *x* − *x* ^*∗*^ − ∫_*x*^*∗*^_^*x*^(*f*(*x* ^*∗*^, *v* ^*∗*^)/*f*(*s*, *v* ^*∗*^))*ds* → +*∞*, as *x* → +*∞* or *x* → 0^+^.

The main purpose of this paper is to carry out a complete theoretical analysis on the global stability of the equilibria of system (2). The organization of this paper is as follows. In Section 2, we consider the nonnegativity and boundedness of the solutions and the existence of the equilibria of system (2). In Section 3, we consider the global stability of the infection-free equilibrium *E*_0_ and the infected equilibrium without immunity *E*_1_ by constructing suitable Lyapunov functionals and using LaSalle invariance principle. In Section 4, we discuss the local stability of the infected equilibrium with CTL response *E* ^*∗*^ and the existence of Hopf bifurcations. Finally, in Section 5, the brief conclusions are given and some numerical simulations are carried out to illustrate the main results.

## 2. Basic Results

### 2.1. The Nonnegativity and Boundedness of the Solutions

According to biological meanings, the initial condition of system (2) is given as follows:

(3) xθφ1θ, uθ=φ2θ, yθ=φ3θ,vθφ4θ, zθ=φ5θ,

where *θ* ∈ [−*τ*, 0] and (*φ*_1_, *φ*_2_, *φ*_3_, *φ*_4_, *φ*_5_) ∈ *C* = *C*([−*τ*, 0], *R*_+_^5^) and *C* is the Banach space of the continuous functions mapping the interval [−*τ*, 0] into *R*_+_^5^, *R*_+_^5^ = {(*x*_1_, *x*_2_, *x*_3_, *x*_4_, *x*_5_)∣*x*_*i*_ ≥ 0, *i* = 1,2, 3,4, 5}.

Under the initial condition (3), it easily shows that the solution of system (2) is unique and nonnegative for all *t* ≥ 0 and ultimately bounded. It has the following result.

**Proposition 1 .**

Under the initial condition (3), the solution of system (2) is unique and nonnegative for all *t* ≥ 0 and also ultimately bounded, when (*H1*)–(*H7*) are satisfied.

**Proof**

The uniqueness and nonnegativity of the solution (*x*(*t*), *u*(*t*), *y*(*t*), *v*(*t*), *z*(*t*)) can be easily proved by using the theorems in [34, 35].

Next, for *t* ≥ 0, define

(4) $$\begin{array}{c} L\left(\;t\;\right)=x\left(\;t\;\right)+u\left(\;t\;\right)+y\left(\;t\;\right)+\frac{\mu_{3}}{2k}v\left(\;t\;\right)+\frac{p}{2q}z\left(\;t+\tau \;\right). \end{array}$$

By the nonnegativity of the solutions, it follows that, for *t* ≥ 0,

(5) L′tλ−μ1xt−μ2ut−μ32yt−μ3μ42kvt−pμ52qzt+τ−p2ytzt−pm2qyt+τzt+τ≤λ−γLt,

where *γ* = min{*μ*_1_, *μ*_2_, *μ*_3_/2, *μ*_4_, *μ*_5_}. Thus, it has that limsup_*t*→+*∞*_ *L*(*t*) ≤ *λ*/*γ*, from which it has that the solution (*x*(*t*), *u*(*t*), *y*(*t*), *v*(*t*), *z*(*t*)) is ultimately bounded.

### 2.2. The Existence of the Equilibria

Next, we consider the existence of the equilibria. The equilibrium of system (2) satisfies

(6) λ−fx,vv−μ1x=0,fx,vv−σ+μ2u=0,σu−pyz−μ3y=0,ky−μ4v=0,qyz−μ5z−myz=0.

If *u* = 0, *y* = 0, *v* = 0, and *z* = 0, system (2) has only one equilibrium, that is, the infection-free equilibrium *E*_0_ = (*x*_0_, 0,0, 0,0), where *x*_0_ = *λ*/*μ*_1_.

If *u* ≠ 0, *y* ≠ 0, *v* ≠ 0, and *z* = 0, we have

(7) fx,kσλ−μ1xμ3μ4σ+μ2−μ3μ4σ+μ2kσ=0,

(8) y=σλ−μ1xμ3σ+μ2,

Since *v* > 0, we have that *x* < *λ*/*μ*_1_. Hence, we only need to consider the case of *x* < *λ*/*μ*_1_.

Consider the following function defined on the interval (0, *λ*/*μ*_1_) by

(9) $$\begin{array}{c} F\left(\;x\;\right)=f\left(\;x,\frac{k\sigma \left(\lambda -\mu_{1}x\right)}{\mu_{3}\mu_{4}\left(\sigma +\mu_{2}\right)}\;\right)-\frac{\mu_{3}\mu_{4}\left(\sigma +\mu_{2}\right)}{k\sigma }. \end{array}$$

Under hypotheses (H2) and (H3), we have

(10) $$\begin{array}{c} F^{\prime }\left(\;x\;\right)=\frac{\partial f}{\partial x}+\frac{\partial f}{\partial v}\left(\;\frac{-k\sigma \mu_{1}}{\mu_{3}\mu_{4}\left(\sigma +\mu_{2}\right)}\;\right)>0. \end{array}$$

We know that the function *F*(*x*) is strictly monotonically increasing with respect to *x*. Denote the basic reproduction number *R*_0_ of system (2) by

(11) $$\begin{array}{c} R_{0}=\frac{k\sigma f\left(\lambda /\mu_{1},0\right)}{\mu_{3}\mu_{4}\left(\sigma +\mu_{2}\right)}. \end{array}$$

Clearly, we have

(12) F0−μ3μ4σ+μ2kσ<0,Fλμ1fλμ1,0−μ3μ4σ+μ2kσ=μ3μ4σ+μ2kσR0−1.

It has that there exists a unique *x*_1_ ∈ (0, *λ*/*μ*_1_) such that *F*(*x*_1_) = 0, if *R*_0_ > 1. Then we can compute *u*_1_, *y*_1_ and *v*_1_ by (8). Hence, we get the unique infected equilibrium without immunity *E*_1_ = (*x*_1_, *u*_1_, *y*_1_, *v*_1_, 0).

If *z* ≠ 0 and *q* > *m*, we get the following equations:

(13) fx,kμ5μ4q−mkμ5μ4q−m−λ+μ1x=0,

(14) u=λ−μ1xσ+μ2,

(15) z=λ−μ1xq−mσ−μ3μ5σ+μ2pμ5σ+μ2.

Since *z* > 0, we have $x<\bar{x}$, where

(16) $$\begin{array}{c} \bar{x}=\frac{\lambda \left(q-m\right)\sigma -\mu_{3}\mu_{5}\left(\sigma +\mu_{2}\right)}{\mu_{1}\left(q-m\right)\sigma }. \end{array}$$

Hence, the existence of the equilibrium requires $\bar{x}>0$ and (13) has a solution on the interval $\left(0,\bar{x}\right)$.

Denote

(17) $$\begin{array}{c} \bar{R}=\frac{\lambda \left(q-m\right)\sigma }{\mu_{3}\mu_{5}\left(\sigma +\mu_{2}\right)}. \end{array}$$

Hence, if $\bar{R}>1$, it has $\bar{x}>0$. Denote

(18) $$\begin{array}{c} G\left(\;x\;\right)=f\left(\;x,\frac{k\mu_{5}}{\mu_{4}\left(q-m\right)}\;\right)\frac{k\mu_{5}}{\mu_{4}\left(q-m\right)}-\lambda +\mu_{1}x. \end{array}$$

Under hypothesis (H2), we know that the function *G*(*x*) is strictly monotonically increasing with respect to *x*. Clearly, we have

(19) G0−λ<0,Gx¯fx¯,kμ5μ4q−mkμ5μ4q−m−λ+μ1x¯=fx¯,kμ5μ4q−mkμ5μ4q−m−μ3μ5σ+μ2q−mσ=μ3μ5σ+μ2q−mσR1−1,

where

(20) $$\begin{array}{c} R_{1}=\frac{k\sigma f\left(\bar{x},k\mu_{5}/\mu_{4}\left(q-m\right)\right)}{\mu_{3}\mu_{4}\left(\sigma +\mu_{2}\right)}. \end{array}$$

Hence, we have that there exists $x^{*}\in (0,\bar{x})$ such that *G*(*x* ^*∗*^) = 0, if $\bar{R}>1$ and *R*_1_ > 1. Then we can compute *u* ^*∗*^, *y* ^*∗*^, *v* ^*∗*^, and *z* ^*∗*^ by (14) and (15).

Denote the immune response reproduction number of system (2) as *R*_1_. Therefore, we have that there exists a unique infected equilibrium with CTL response *E* ^*∗*^ = (*x* ^*∗*^, *u* ^*∗*^, *y* ^*∗*^, *v* ^*∗*^, *z* ^*∗*^), if $\bar{R}>1$ and *R*_1_ > 1. This proves the following theorem.

**Theorem 2 .**

Suppose that hypotheses (H1)–(H4) are satisfied; the following conclusions hold.

(i) System (2) always has an infection-free equilibrium *E*_0_.
(ii) System (2) has an infected equilibrium without immunity *E*_1_ if *R*_0_ > 1.
(iii) System (2) has an infected equilibrium with immunity *E* ^*∗*^ if $\bar{R}>1$ and *R*_1_ > 1.

From hypotheses (H1)–(H3), it is clear that *R*_1_ < *R*_0_. In order to study the global stability of the infected equilibrium *E*_1_ in the next section, we give the following remark.

**Remark 3 .**

Suppose that $\bar{R}>1$ is satisfied; then the following results hold:

(i) If *R*_1_ > 1, then (*q* − *m*)*y*_1_/*μ*_5_ > 1.
(ii) If *R*_1_ ≤ 1, then (*q* − *m*)*y*_1_/*μ*_5_ ≤ 1.

Let us give the proof of Remark 3. Firstly, for Case (i), since *R*_1_ > 1, then

(21) Fx¯fx¯,kσλ−μ1x¯μ3μ4σ+μ2−μ3μ4σ+μ2kσ=μ3μ4σ+μ2kσR1−1>0.

Since the function *F*(*x*) is strictly monotonically increasing with respect to *x* and *F*(*x*_1_) = 0, we have $x_{1}<\bar{x}$. Therefore

(22) $$\begin{array}{c} \lambda -\mu_{1}x_{1}>\lambda -\mu_{1}\bar{x}=\frac{\mu_{3}\mu_{5}\left(\sigma +\mu_{2}\right)}{\sigma \left(q-m\right)}. \end{array}$$

Then

(23) $$\begin{array}{c} \frac{\left(q-m\right)y_{1}}{\mu_{5}}=\frac{q-m}{\mu_{5}}\cdot \frac{\sigma \left(\lambda -\mu_{1}x_{1}\right)}{\mu_{3}\left(\sigma +\mu_{2}\right)}>1. \end{array}$$

Secondly, for Case (ii), since *R*_1_ ≤ 1, then

(24) Fx¯fx¯,kσλ−μ1x¯μ3μ4σ+μ2−μ3μ4σ+μ2kσ=μ3μ4σ+μ2kσR1−1≤0.

We have $x_{1}\geq \bar{x}$. Therefore

(25) $$\begin{array}{c} \lambda -\mu_{1}x_{1}\leq \lambda -\mu_{1}\bar{x}=\frac{\mu_{3}\mu_{5}\left(\sigma +\mu_{2}\right)}{\sigma \left(q-m\right)}. \end{array}$$

Then

(26) $$\begin{array}{c} \frac{\left(q-m\right)y_{1}}{\mu_{5}}=\frac{q-m}{\mu_{5}}\cdot \frac{\sigma \left(\lambda -\mu_{1}x_{1}\right)}{\mu_{3}\left(\sigma +\mu_{2}\right)}\leq 1. \end{array}$$

## 3. The Global Stability of the Equilibria

In this section, we study the global stability of the equilibria of system (2). Firstly, we analyze the global stability of the infection-free equilibrium *E*_0_.

**Theorem 4 .**

Suppose that hypotheses (H1)–(H7) are satisfied. If *R*_0_ ≤ 1, then the infection-free equilibrium *E*_0_ is globally asymptotically stable for any time delay *τ* ≥ 0. If *R*_0_ > 1, then the infection-free equilibrium *E*_0_ is unstable for any time delay *τ* ≥ 0.

**Proof**

Let (*x*(*t*), *u*(*t*), *y*(*t*), *v*(*t*), *z*(*t*)) be a positive solution of system (2) with the initial condition (3) for *t* ≥ 0. Motivated by the works in [14, 28, 31, 36, 37], we consider the following Lyapunov functional:

(27) V1=x−x0−∫x0xfx0,0fs,0ds+u+σ+μ2σy+μ3σ+μ2kσv+σ+μ2σpq−mz+σ+μ2σpq−m∫t−τtqyθzθdθ,

where *λ* = *μ*_1_ *x*_0_. By (H1)–(H5), it is obvious that *V*_1_ is positive definite with respect to *E*_0_. For *t* ≥ 0, the time derivative of *V*_1_ along the solutions of system (2) is

(28) V˙1=1−fx0,0fx,0x˙+u˙+σ+μ2σy˙+μ3σ+μ2kσv˙+σ+μ2σpq−mz˙+σ+μ2σpq−mqytzt−qyt−τzt−τ=μ11−fx0,0fx,0x0−x+fx0,0fx,0fx,vv−μ3μ4σ+μ2kσv−σ+μ2σpq−mμ5z=μ11−fx0,0fx,0x0−x−μ3μ4σ+μ2kσ1−fx,vfx,0R0v−σ+μ2σpq−mμ5z.

Since hypotheses (H1)–(H3) and *R*_0_ ≤ 1, we have

(29) μ11−fx0,0fx,0x0−x≤0,1−fx,vfx,0R0≥0.

Therefore, ${\dot{V}}_{1}\leq 0$ if *R*_0_ ≤ 1. Then it follows from stability theorems in [34, 35] that the infection-free equilibrium *E*_0_ is stable for any time delay *τ* ≥ 0 if *R*_0_ ≤ 1.

Furthermore, note that, for each *t* ≥ 0, ${\dot{V}}_{1}=0$ implies that *x*(*t*) = *x*_0_, *z*(*t*) = 0. Let *M* be the largest invariant set in the set

(30) Γ1=φ1,φ2,φ3,φ4,φ5∈C ∣ V˙1=0⊂φ1,φ2,φ3,φ4,φ5∈C ∣ φ10=x0,  φ50=0.

We have from the first four equations of system (2) and the invariance of *M* that *M* = {*E*_0_}. Since any solution of system (2) is bounded, it follows from LaSalle invariance principle (see, e.g., [34, 35]) that the infection-free equilibrium *E*_0_ is also globally attractive for any time delay *τ* ≥ 0 if *R*_0_ ≤ 1.

The characteristic equation of system (2) at the infection-free equilibrium *E*_0_ is
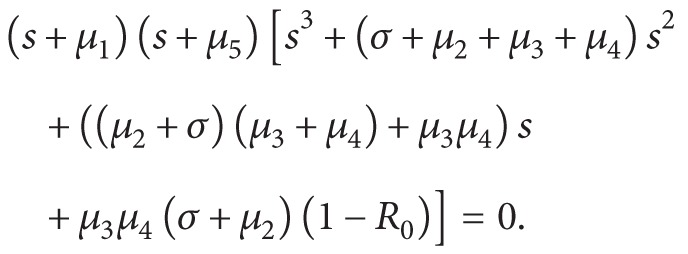
(31)Clearly, if *R*_0_ > 1, (31) has at least a positive real root. Thus, the infection-free equilibrium *E*_0_ is unstable.

Next we study the global stability of the infected equilibrium without immunity *E*_1_.

**Theorem 5 .**

Suppose that hypotheses (H1)–(H7) and $\bar{R}>1$ are satisfied. If *R*_0_ > 1 ≥ *R*_1_, then the infected equilibrium without immunity *E*_1_ is globally asymptotically stable for any time delay *τ* ≥ 0. If *R*_1_ > 1, then the infected equilibrium without immunity *E*_1_ is unstable for any time delay *τ* ≥ 0.

**Proof**

Let (*x*(*t*), *u*(*t*), *y*(*t*), *v*(*t*), *z*(*t*)) be a positive solution of system (2) with the initial condition (3) for *t* ≥ 0. Consider the following Lyapunov functional:

(32) V2=x−x1−∫x1xfx1,v1fs,v1ds+u−u1−u1ln⁡uu1+σ+μ2σy−y1−y1ln⁡yy1+μ3σ+μ2kσv−v1−v1ln⁡vv1+σ+μ2σpq−mz+σ+μ2σpqq−m∫t−τtyθzθdθ.

Let *ψ*(*x*) = *x* − *x*_1_ − ∫_*x*_1__^*x*^(*f*(*x*_1_, *v*_1_)/*f*(*s*, *v*_1_))*ds*. Then, *ψ*(*x*) has the global minimum at *x* = *x*_1_ and *ψ*(*x*_1_) = 0. Furthermore, *ψ*(*x*) > 0 for *x* > 0. Hence, *V*_2_ is positive definite with respect to *E*_1_. For *t* ≥ 0, the time derivative of *V*_2_ along the solutions of system (2) is

(33) V˙2=1−fx1,v1fx,v1x˙+1−u1uu˙+σ+μ2σ1−y1yy˙+μ3σ+μ2kσ1−v1vv˙+σ+μ2σpq−mz˙+σ+μ2σ·qq−mpytzt−pyt−τzt−τ=1−fx1,v1fx,v1λ−fx,vv−μ1x+1−u1ufx,vv−σ+μ2u+σ+μ2σ1−y1yσu−pyz−μ3y+μ3σ+μ2kσ1−v1vky−μ4v+σ+μ2σ·pq−mqyt−τzt−τ−μ5z−myz+σ+μ2σ·qq−mpytzt−pyt−τzt−τ.

Note that *λ* = *f*(*x*_1_, *v*_1_)*v*_1_ + *μ*_1_ *x*_1_, *f*(*x*_1_, *v*_1_)*v*_1_ = (*σ* + *μ*_2_)*u*_1_, and *μ*_3_ *y*_1_ = *σu*_1_; we have

(34) V˙2=1−fx1,v1fx,v1fx1,v1v1+μ1x1−fx,vv−μ1x+1−u1ufx,vv−fx1,v1v1u1u+σ+μ2σ1−y1y−pyz+fx1,v1v11−y1yuu1−yy1+μ3σ+μ2kσ1−v1vky−μ4v+σ+μ2σ·pq−mqyt−τzt−τ−μ5z−myz+σ+μ2σqq−mpytzt−pyt−τzt−τ=μ11−fx1,v1fx,v1x1−x+σ+μ2σpzy1−μ5q−m+fx1,v1v15−y1yuu1−fx,vvfx1,v1v1u1u−yy1v1v−fx1,v1fx,v1−fx,v1fx,v+fx1,v1v1−1−vv1+fx,vvfx,v1v1+fx,v1fx,v.

Since the arithmetic mean is greater than or equal to the geometric mean, it has

(35) 5−y1yuu1−fx,vvfx1,v1v1u1u−yy1v1v−fx1,v1fx,v1−fx,v1fx,v≤0.

From hypotheses (H3)-(H4), we have

(36) −1−vv1+fx,vvfx,v1v1+fx,v1fx,v=fx,v−fx,v1fx,v1fx,vv−fx,v1v1fx,vv1≤0.

Note Remark 3, we have *y*_1_ ≤ *μ*_5_/(*q* − *m*). Therefore, ${\dot{V}}_{2}\leq 0$ if *R*_1_ ≤ 1. Then it follows from stability theorems in [34, 35] that the infected equilibrium without immunity *E*_1_ is stable for any time delay *τ* ≥ 0 if *R*_1_ ≤ 1.

Furthermore, note that, for each *t* ≥ 0, ${\dot{V}}_{2}=0$ implies that *x*(*t*) = *x*_1_, *u*(*t*) = *u*_1_, *y*(*t*) = *y*_1_, and *v*(*t*) = *v*_1_. Let *M* be the largest invariant set in the set

(37) Γ2=φ1,φ2,φ3,φ4,φ5∈C ∣ V˙2=0⊂φ1,φ2,φ3,φ4,φ5∈C ∣ φ10=x1,  φ20=u1,  φ30=y1,  φ40=v1.

We have from system (2) and the invariance of *M* that *M* = {*E*_1_}. Since any solution of system (2) is bounded, it follows from LaSalle invariance principle (see, e.g., [34, 35]) that the infected equilibrium without immunity *E*_1_ is also globally attractive for any time delay *τ* ≥ 0 if *R*_1_ ≤ 1.

The characteristic equation of system (2) at *E*_1_ takes the form

(38) $$\begin{array}{c} \left(\;s+\mu_{5}+my_{1}-qy_{1}e^{-s\tau }\;\right)\psi_{0}\left(\;s\;\right)=0, \end{array}$$

where *ψ*_0_(*s*) is a polynomial with respect to *s*. Let

(39) $$\begin{array}{c} \psi_{1}\left(\;s\;\right)=s+\mu_{5}+my_{1}-qy_{1}e^{-s\tau }. \end{array}$$

Thus we have lim_*s*→+*∞*_ *ψ*_1_(*s*) > 0 and *ψ*_1_(0) = *μ*_5_ − (*q* − *m*)*y*_1_. From Remark 3, we have that (*q* − *m*)*y*_1_/*μ*_5_ > 1 if *R*_1_ > 1. Thus, *ψ*_1_(0) < 0 if *R*_1_ > 1. Hence, if *R*_1_ > 1, then *ψ*_1_(*s*) = 0 has at least a positive real root; that is, (38) has at least a positive real root. Therefore, the infected equilibrium without immunity *E*_1_ is unstable.

## 4. The Local Stability of the Infected Equilibrium and Hopf Bifurcation

The characteristic equation of system (2) at the infected equilibrium with CTL response *E* ^*∗*^ is given by

(40) s5+A1s4+A2s3+A3s2+A4s+A5+e−sτB1s4+B2s3+B3s2+B4s+B5=0,

where

(41) A1=A+D+E+μ4+qy∗,A2=E+μ4qy∗+μ4E+DE+μ4+qy∗+AD+E+μ4+qy∗−py∗mz∗,A3=μ4Eqy∗+Dμ4+Eqy∗−σkB+Aμ4+Eqy∗+μ4E+DE+μ4+qy∗−py∗mz∗A+D+μ4,A4=−Bkσqy∗+Aμ4Eqy∗+Dμ4+Eqy∗−Bkσ+CkσB+H−py∗mz∗Dμ4+Aμ4+AD,A5=CσkB+Hqy∗−py∗mz∗ADμ4−ABσkqy∗,B1=−qy∗,B2=−qy∗A+D+μ3+μ4,B3=−qy∗μ3μ4+Dμ3+μ4+AD+μ3+μ4,B4=−qy∗Dμ3μ4+Aμ3μ4+Dμ3+Dμ4−σkB+H,B5=−qy∗ADμ3μ4−AσkB+H+CσkB+H,a=∂fx∗,v∗∂x>0,d=∂fx∗,v∗∂v≤0,A=av∗+μ1,B=dv∗,C=av∗,D=σ+μ2,E=μ3+pz∗,F=q−mz∗,H=fx∗,v∗,M=py∗F.

When *τ* = 0, (40) becomes

(42) $$\begin{array}{c} s^{5}+\alpha_{1}s^{4}+\alpha_{2}s^{3}+\alpha_{3}s^{2}+\alpha_{4}s+\alpha_{5}=0, \end{array}$$

where

(43) α1A1+B1=A+D+E+μ4>0,α2A2+B2=M+AD+E+μ4+Eμ4+Dμ4+DE>0,α3A3+B3=A+D+μ4M+AEμ4+Dμ4+DE−Bkσ>0,α4A4+B4=Dμ4+Aμ4+ADM+G>0,α5A5+B5=ADμ4M>0,GCDEμ4−μ1Bkσ>0.

Denote

(44) Δ1=α1,Δ2=α1α2−α3,Δ3=α3Δ2+α1α5−α12α4,Δ4=α4Δ3+α1α5−α2α5Δ2−α52,Δ5=α5Δ4.

Since *μ*_4_(*σ* + *μ*_2_)(*μ*_3_ + *pz* ^*∗*^) = *σkf*(*x* ^*∗*^, *v* ^*∗*^) and (H4), we have *σkH* = *μ*_4_ *DE*, *μ*_4_ *DE* + *Bkσ* > 0, and *ADEμ*_4_ − *G* > 0.

Thus,

(45) Δ1=α1>0,Δ2=EM+A2D+E+μ4+D+E+μ4AD+E+μ4+DE+μ4+Eμ4+Bkσ>0,Δ3=MAA2E+AE2+E2μ4+DEμ4+EM+Bkσ+DA2E+D2E+ADE+AE2+DE2+DEμ4+EM+Bkσ+μ4A2E+D2E+DEμ4+ADE+AE2+DE2+DEμ4+E2μ4+Eμ42+EM+Bkσ+μ1DEμ4+BkσA+D+E+μ42+ADEA2D+A2E+AD2+ADE+D2E+DEμ4+ADE+AE2+DE2+EM+Bkσ+ADμ4A2D+A2E+A2μ4+AD2+ADE+ADμ4+D2E+D2μ4+ADE+AE2+AEμ4+DE2+DEμ4+ADμ4+Aμ42+DEμ4+Dμ42+EM+Bkσ+AEμ4−BkσΔ2>0,Δ4=N1M3+N2M2+N3M+N4,

where 
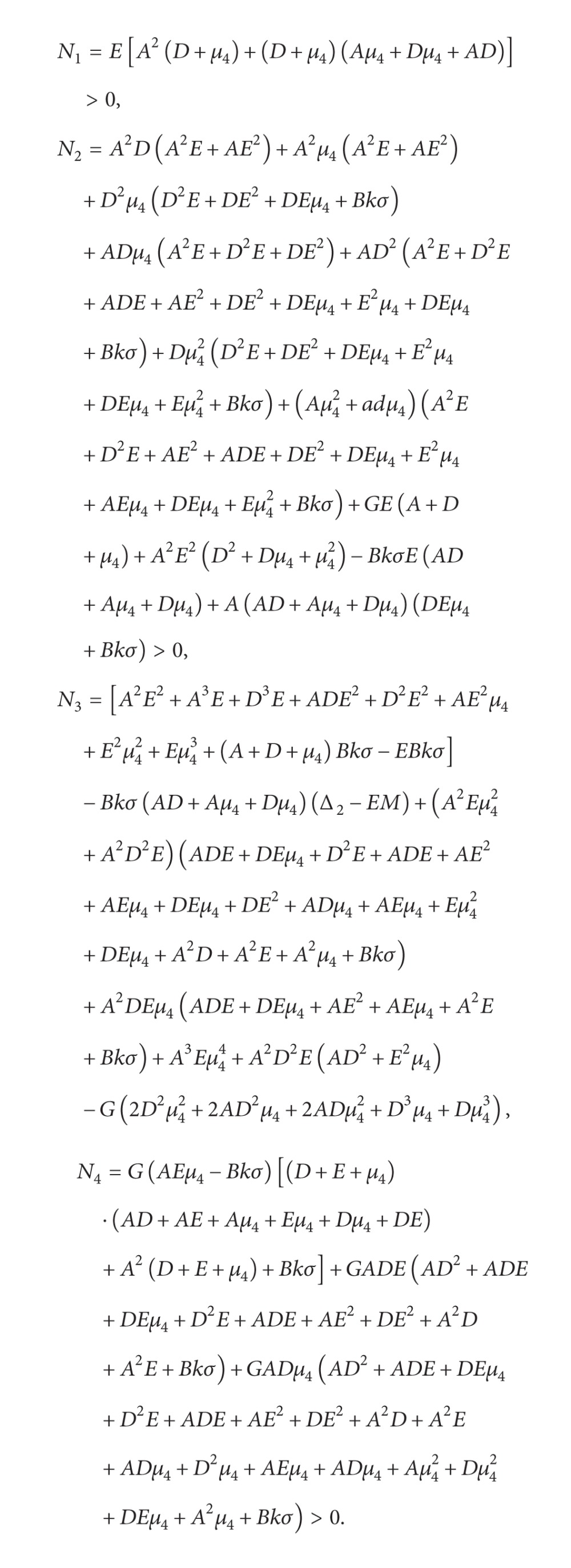
(46)


Assume further that

(H8) *E* ≥ max{*μ*_4_, *D*}; that is, *μ*_3_ + *pz* ^*∗*^ ≥ *μ*_4_ and *μ*_3_ + *pz* ^*∗*^ ≥ *σ* + *μ*_2_.

We have 
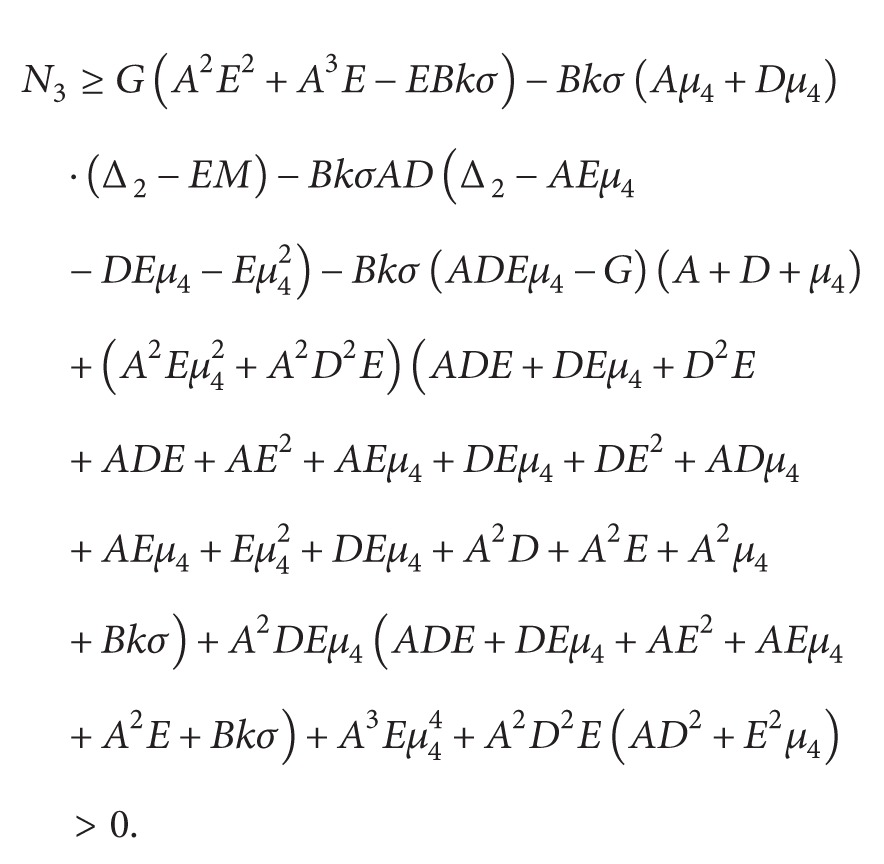
(47)Therefore, Δ_4_ > 0, Δ_5_ > 0. By Routh-Hurwitz criterion, all the roots of (42) have negative real parts. Hence we have the following result.

**Proposition 6 .**

When *τ* = 0, if $\bar{R}>1$, *R*_1_ > 1, and (H8) hold, then the infected equilibrium with CTL response *E* ^*∗*^ is locally asymptotically stable.

In fact, when *τ* = 0, we can show that if $\bar{R}>1$ and *R*_1_ > 1 hold, the infected equilibrium with CTL response *E* ^*∗*^ is globally asymptotically stable by constructing suitable Lyapunov function.

**Proposition 7 .**

Suppose that hypotheses (*H1*)–(*H7*) and $\bar{R}>1$ are satisfied. If *R*_1_ > 1, then the infected equilibrium with CTL response *E* ^*∗*^ is globally asymptotically stable when *τ* = 0.

**Proof**

By the following Lyapunov function,

(48) V3=x−x∗−∫x∗xfx∗,v∗fs,v∗ds+u−u∗−u∗ln⁡uu∗+σ+μ2σy−y∗−y∗ln⁡yy∗+fx∗,v∗μ4v−v∗−v∗ln⁡vv∗+σ+μ2σpq−mz−z∗−z∗ln⁡zz∗,

*V*_3_ is positive definite with respect to *E* ^*∗*^. For *t* ≥ 0, the time derivative of *V*_3_ along the solutions of system (2) is

(49) V˙31−fx∗,v∗fx,v∗x˙+1−u∗uu˙+σ+μ2σ1−y∗yy˙+fx∗,v∗μ41−v∗vv˙+σ+μ2σpq−m1−z∗zz˙=1−fx∗,v∗fx,v∗λ−fx,vv−μ1x+1−u∗ufx,vv−σ+μ2u+σ+μ2σ1−y∗yσu−pyz−μ3y+fx∗,v∗μ41−v∗vky−μ4v+σ+μ2σpq−m1−z∗zqyz−μ5z−myz.

Note that *λ* = *f*(*x* ^*∗*^, *v* ^*∗*^)*v* ^*∗*^ + *μ*_1_ *x* ^*∗*^, *f*(*x* ^*∗*^, *v* ^*∗*^)*v* ^*∗*^ = (*σ* + *μ*_2_)*u* ^*∗*^, and *μ*_3_ *y* ^*∗*^ = *σu* ^*∗*^ − *py* ^*∗*^ *z* ^*∗*^; we have

(50) V˙3=1−fx∗,v∗fx,v∗fx∗,v∗v∗+μ1x∗−fx,vv−μ1x+1−u∗ufx,vv−fx∗,v∗v∗u∗u+σ+μ2σ1−y∗ypyz∗−pyz+fx∗,v∗v∗σu∗1−y∗yσu−σu∗yy∗+fx∗,v∗μ41−v∗vky−μ4v+σ+μ2σ·pq−m1−z∗zqyz−μ5z−myz=μ1x∗−x1−fx∗,v∗fx,v∗+fx∗,v∗·v∗−1+fx,vfx,v∗vv∗−vv∗+fx,v∗fx,v+fx∗,v∗·v∗5−fx∗,v∗fx,v∗−fx,vfx∗,v∗vv∗u∗u−y∗yuu∗−fx,v∗fx,v−yy∗v∗v.

Since the arithmetic mean is greater than or equal to the geometric mean, it has

(51) 5−fx∗,v∗fx,v∗−fx,vfx∗,v∗vv∗u∗u−y∗yuu∗−fx,v∗fx,v−yy∗v∗v≤0.

From hypotheses (H3)-(H4), we have

(52) −1+fx,vfx,v∗vv∗−vv∗+fx,v∗fx,v=fx,v−fx,v∗fx,v∗fx,vv−fx,v∗v∗fx,vv∗≤0.

Therefore, ${\dot{V}}_{3}\leq 0$ if *R*_1_ > 1. Then it follows from stability theorems in [34, 35] that the infected equilibrium CTL response *E* ^*∗*^ is stable for *τ* = 0 if *R*_1_ > 1. Similarly, by LaSalle invariance principle, we can show that the infected equilibrium CTL response *E* ^*∗*^ is also globally attractive for *τ* = 0 if *R*_1_ > 1.

Next, we consider the case when *τ* > 0. Since *α*_5_ > 0, *s* = 0 is not a root of (40). We suppose (40) has a purely imaginary root *s* = *iω*  (*ω* > 0) for some *τ* > 0. Substituting *s* = *iω* into (40) and separating the real and imaginary parts, we have

(53) ω5−A2ω3+A4ω=B1ω4−B3ω2+B5sin⁡ωτ+B2ω3−B4ωcos⁡ωτ,A1ω4−A3ω2+A5=−B1ω4−B3ω2+B5cos⁡ωτ+B2ω3−B4ωsin⁡ωτ.

Squaring and adding the two equations of (53), it follows that

(54) $$\begin{array}{c} \omega^{10}+C_{1}\omega^{8}+C_{2}\omega^{6}+C_{3}\omega^{4}+C_{4}\omega^{2}+C_{5}=0, \end{array}$$

where

(55) C1=A12−2A2−B12,C2=A22+2A4−2A1A3+2B1B3−B22,C3=A32−2A2A4−B32+2B2B4+2A1A5−2B1B5,C4=A42−B42−2A3A5+2B3B5,C5=A52−B52.

Letting *ν* = *ω* ^2^, (54) can be written as

(56) $$\begin{array}{c} h\left(\;\nu \;\right)=\nu^{5}+C_{1}\nu^{4}+C_{2}\nu^{3}+C_{3}\nu^{2}+C_{4}\nu +C_{5}=0. \end{array}$$

Then we have

(57) $$\begin{array}{c} h^{\prime }\left(\;\nu \;\right)=5\nu^{4}+4C_{1}\nu^{3}+3C_{2}\nu^{2}+2C_{3}\nu +C_{4}. \end{array}$$

Denote

(58) p1=−625C12+35C2,q1=8125C13−625C1C2+25C3,r1=−3625C14+3125C12C2−225C1C3+15C4,Θ0=p12−4r1,p2=−13p12−4r1,q2=−227p13+83p1r1−q12,Θ1=127p23+14q22,s∗=−q22+Θ13+−q22−Θ13+13p1,Θ2=−s∗−p1+2q1s∗−p1,Θ3=−s∗−p1−2q1s∗−p1.

By a similar argument as that in [38], we have the following results.

**Lemma 8 .**

For the polynomial equation (56), the following results hold.

(i) Equation (56) has at least one positive root, if one of the following conditions (*a*)–(*d*) holds:
  (a) *C*_5_ < 0.
  (b) *C*_5_ ≥ 0, *q*_1_ = 0, Θ_0_ ≥ 0, and *p*_1_ < 0 or *r*_1_ ≤ 0 and there exists *ν* ^*∗*^ ∈ {*ν*_1_, *ν*_2_, *ν*_3_, *ν*_4_} such that *ν* ^*∗*^ > 0 and *h*(*ν* ^*∗*^) ≤ 0, where *ν*_*i*_ = *y*_*i*_ − (1/5)*C*_1_  (*i* = 1,2, 3,4), and
    (59) y1=−p1+Θ02,y2=−−p1+Θ02,y3=−p1−Θ02,y4=−−p1−Θ02.
  (c) *C*_5_ ≥ 0, *q*_1_ ≠ 0, *s*_*∗*_ > *p*_1_, Θ_2_ ≥ 0, or Θ_3_ ≥ 0 and there exists *ν* ^*∗*^ ∈ {*ν*_1_^*∗*^, *ν*_2_^*∗*^, *ν*_3_^*∗*^, *ν*_4_^*∗*^} such that *ν* ^*∗*^ > 0 and *h*(*ν* ^*∗*^) ≤ 0, where *ν*_*i*_ = *y*_*i*_ − (1/5)*C*_1_  (*i* = 1,2, 3,4), and
    (60) y1=−s∗−p1+Θ22,y2=−s∗−p1−Θ22,y3=s∗−p1+Θ32,y4=s∗−p1−Θ32.
  (d) $C_{5}\geq 0,\;\;q\neq 0,\;\;s_{*}<p_{1},\;\;q_{1}^{2}/4{\left(p_{1}-s_{*}\right)}^{2}+\left(1/2\right)s_{*}=0,\;\;\bar{\nu }>0$, and $h(\bar{\nu })\leq 0$, where $\bar{\nu }=q_{1}/2(p_{1}-s_{*})-\left(1/5\right)C_{1}$.
(ii) If the conditions (a)–(d) of (*i*) are all not satisfied, then (56) has no positive real root.

Suppose that *h*(*ν*) = 0 has positive real roots. Without loss of generality, we may assume that (56) has $\bar{k}\;\;(1\leq \bar{k}\leq 5)$ positive real roots, denoted, respectively, as $\nu_{1},\nu_{2},\ldots ,\nu_{\bar{k}}$. Then, (54) has positive real roots $\omega_{\bar{k}}=\sqrt{v_{\bar{k}}}$. From (40), we get

(61) $$\begin{array}{c} \cos\omega \tau =\frac{\left(\omega^{5}-A_{2}\omega^{3}+A_{4}\omega \right)\left(B_{2}\omega^{3}-B_{4}\omega \right)-\left(A_{1}\omega^{4}-A_{3}\omega^{2}+A_{5}\right)\left(B_{1}\omega^{4}-B_{3}\omega^{2}+B_{5}\right)}{{\left(B_{2}\omega^{3}-B_{4}\omega \right)}^{2}+{\left(B_{1}\omega^{4}-B_{3}\omega^{2}+B_{5}\right)}^{2}}\equiv L\left(\;\omega \;\right). \end{array}$$

Therefore, let

(62) $$\begin{array}{c} \tau_{k}^{\left(j\right)}=\frac{1}{\omega_{k}}\left\{\;\arccos L\left(\;\omega_{k}\;\right)+2\pi j\;\right\}, \end{array}$$

where $k=1,2,\ldots ,\bar{k},\;\;j=0,1,\ldots$. Then ±*iω*_*k*_ are a pair of purely imaginary roots of (54) with *τ* = *τ*_*k*_^(*j*)^.

Define

(63) τ0τk00=mink∈1,2,…,k¯⁡τk0,ω0ωk0.

Let *s*(*τ*) = *ξ*(*τ*) + *iω*(*τ*) be a root of (40) satisfying *ξ*(*τ*_*k*_^(*j*)^) = 0 and *ω*(*τ*_*k*_^(*j*)^) = *ω*_*k*_. Differentiating the two sides of (40) with respect to *τ* and noticing that *s* is a function of *τ*, it follows that

(64) dsdτ−1=−5s4+4A1s3+3A2s2+2A3s+A4ss5+A1s4+A2s3+A3s2+A4s+A5+4B1s3+3B2s2+2B3s+B4sB1s4+B2s3+B3s2+B4s+B5−τs.

Thus, we get 
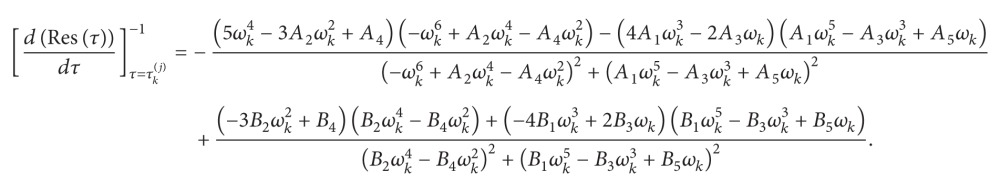
(65)
From (40), we attain

(66) ω5−A2ω3+A4ω2+A1ω4−A3ω2+A52=B2ω3−B4ω2+B1ω4−B3ω2+B52.

Then

(67) dResτdττ=τkj−1=5νk4+4C1νk3+3C2νk2+2C3νk+C4B1ωk4−B3ωk2+B52+B2ωk2−B42ωk2=h′νkB1ωk4−B3ωk2+B52+B2ωk2−B42ωk2.

Therefore, it follows that

(68) sign⁡dResτdττ=τkj=sign⁡dResτdττ=τkj−1=sign⁡h′νk.

Since *ν*_*k*_ > 0, we can know that Re[*ds*_*k*_(*τ*)/*dτ*|_*τ*_ = *τ*_*k*_^(*j*)^] and *h*′(*ν*_*k*_) have the same sign.

From the above analysis, we have the following results.

**Theorem 9 .**

Let *τ*_*k*_^(*j*)^, *τ*_0_, and *ω*_0_ be defined by (62) and (63). If $\bar{R}>1$ and *R*_1_ > 1 are satisfied, then the following results hold:

(i) If the conditions (*a*)–(*d*) of Lemma 8 are all not satisfied, then the infected equilibrium with CTL response *E* ^*∗*^ is locally asymptotically stable for all time delay *τ* > 0.
(ii) If one of the conditions (*a*)–(*d*) of Lemma 8 is satisfied, then the infected equilibrium with CTL response *E* ^*∗*^ is locally asymptotically stable for *τ* ∈ [0, *τ*_0_) and unstable for *τ* > *τ*_0_.
(iii) If all the conditions as stated in (ii) hold and *h*′(*ν*_*k*_) ≠ 0, then system (2) undergoes a Hopf bifurcation at *E* ^*∗*^ when *τ* = *τ*_*k*_^(*j*)^  (*j* = 0,1, 2,…).

## 5. Conclusion and Numerical Simulations

In this paper, we proposed a class of delayed HIV infection model (2) with general incidence rate and immune impairment. This general incidence only satisfies some general hypotheses and includes many types of special incidence functions as special cases. First, we discussed the nonnegativity and boundedness of the solutions and the existence of equilibria of system (2). Then, by constructing suitable Lyapunov functionals and using Lyapunov-LaSalle invariance principle and Hopf bifurcation theorem, we proved the following results.

If *R*_0_ ≤ 1, the infection-free equilibrium *E*_0_ is globally asymptotically stable for any time delay *τ* ≥ 0; that is, any solution (*x*(*t*), *u*(*t*), *y*(*t*), *v*(*t*), *z*(*t*)) → *E*_0_ = (*x*_0_, 0,0, 0,0). In biology, this means that the virus can be finally cleared from the body and the disease dies out. At the same time, as the time *t* increases, the numbers of latently infected cells, actively infected cells, and CTLs trends to zero and the number of uninfected cells trends to a constant *x*_0_.

If *R*_0_ > 1 ≥ *R*_1_ and $\bar{R}>1$, the infected equilibrium without immunity *E*_1_ is globally asymptotically stable for any time delay *τ* ≥ 0; that is, any solution (*x*(*t*), *u*(*t*), *y*(*t*), *v*(*t*), *z*(*t*)) → *E*_1_ = (*x*_1_, *u*_1_, *y*_1_, *v*_1_, 0). In biology, this indicates that the HIV infection will finally become chronic with no persistent CTL response.

If *R*_1_ > 1 and $\bar{R}>1$, there exists a unique infected equilibrium with CTL response *E* ^*∗*^. The result of Theorem 9 implies that the time delay *τ* can destabilize the stability of the infected equilibrium with CTL response *E* ^*∗*^ and leads to the occurrence of Hopf bifurcations.

If the time delay *τ* ∈ [0, *τ*_0_), the infected equilibrium with CTL response *E* ^*∗*^ is locally asymptotically stable. In biology, this implies that the HIV infection may become chronic and the CTL immune response may be persistent. When the time delay *τ* passes through the critical value *τ*_0_, the infected equilibrium with CTL response *E* ^*∗*^ will become unstable and a Hopf bifurcation occurs under some conditions. In biology, this suggests that as the time delay *τ* increases, the numbers of the uninfected cells, latently infected cells, actively infected cells, free virus, and CTLs will first attend constant values and then become oscillated.

We now give numerical simulations to illustrate the main results in Sections 3 and 4.

Let us choose *f*(*x*, *v*)*v* = *βxv*. Then we have that *R*_0_ = *kλβσ*/(*μ*_1_ *μ*_3_ *μ*_4_(*σ* + *μ*_2_)) and *R*_1_ = *R*_0_ − *kβμ*_5_/(*μ*_1_ *μ*_4_(*q* − *m*)). Based on the numerical simulations in [12, 19, 21, 22, 27], let us take the following data:

(69) λ270, β=0.001, k=6, m=0.001,p0.04, q=0.025, σ=0.001, μ1=0.02,μ20.1, μ3=0.8, μ4=1.2, μ5=0.05.

**Figure 1:**
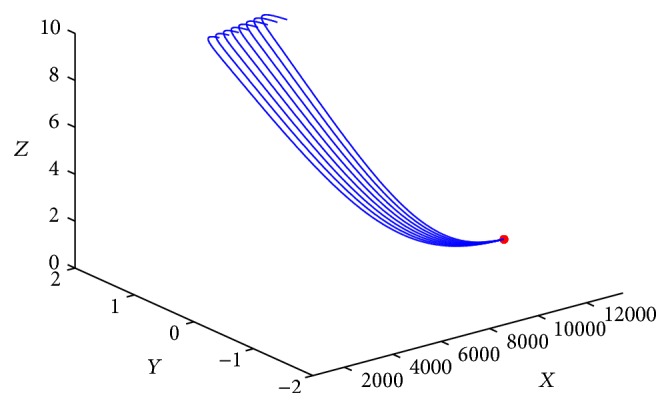
Phase trajectories of system (2) with *R*_0_ ≤ 1.

Direct calculations show that *R*_0_ = 0.8354 < 1 and *R*_1_ = 0.3146 < 1; system (2) has the infection-free equilibrium *E*_0_ = (13500,0, 0,0, 0). By Theorem 4, the infection-free equilibrium *E*_0_ is globally asymptotically stable for any time delay *τ* ≥ 0.  Figure 1 gives the phase trajectories of system (2) with suitable initial condition.

Next, let us choose the following data:

(70) λ270, β=0.001, k=6, m=0.01,p0.04, q=0.025, σ=0.002, μ1=0.02,μ20.1, μ3=0.8, μ4=1.2, μ5=0.05.

**Figure 2:**
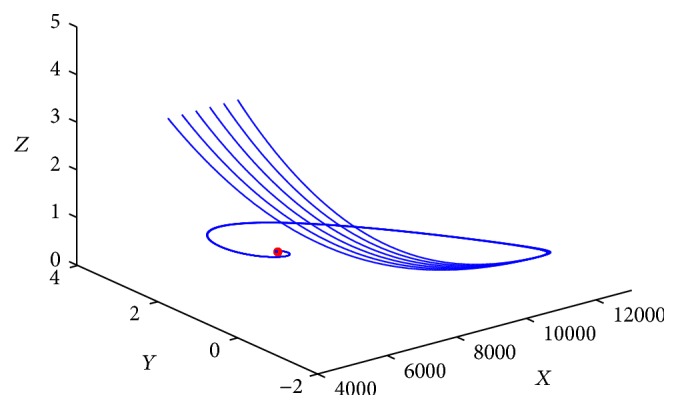
Phase trajectories of system (2) with *R*_0_ > 1 ≥ *R*_1_.

Direct computations show that *R*_0_ = 1.6544 > 1, $\bar{R}=1.9853>1$, and *R*_1_ = 0.8211 < 1; system (2) has the infected equilibrium without immunity *E*_1_ = (8160,1047.0588,2.6176,13.0882,0). Therefore, by Theorem 5, the infected equilibrium without immunity *E*_1_ is globally asymptotically stable for any time delay *τ* ≥ 0.  Figure 2 gives the phase trajectories of system (2) with suitable initial condition.

Furthermore, let us choose the following data:

(71) λ270, β=0.001, k=6, m=0.012,p0.04, q=0.025, σ=0.004, μ1=0.02,μ20.1, μ3=0.8, μ4=1.2, μ5=0.05.

**Figure 3:**
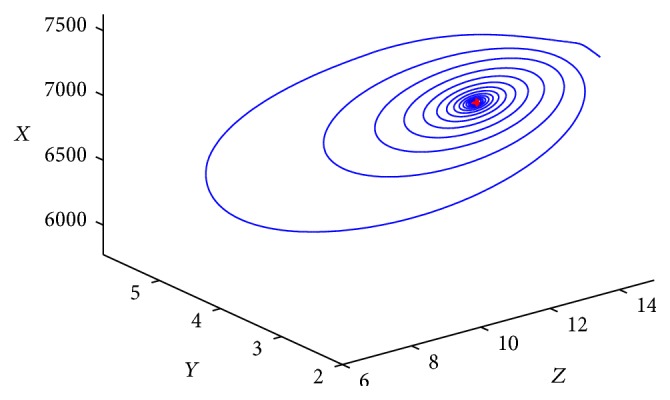
Phase trajectories of system (2) with *R*_1_ > 1, *τ* = 28. The initial condition is (7970, 1000, 2.6, 0.2, 17).

Then we have that *R*_0_ = 3.2452 > 1, $\bar{R}=3.375>1$, and *R*_1_ = 2.2837 > 1 and (56) has no positive root. System (2) has the infected equilibrium with CTL response *E* ^*∗*^ = (6882.3529,1272.6244,3.84615,19.2308,13.0882). From Theorem 9(i), the infected equilibrium with CTL response *E* ^*∗*^ is locally asymptotically stable for any time delay *τ* > 0.  Figure 3 gives the phase trajectories of system (2) with suitable initial condition.

Finally, let us choose the following data:

(72) λ270, β=0.001, k=6, m=0.01,p0.04, q=0.025, σ=0.004, μ1=0.02,μ20.1, μ3=0.8, μ4=1.2, μ5=0.05.

**Figure 4:**
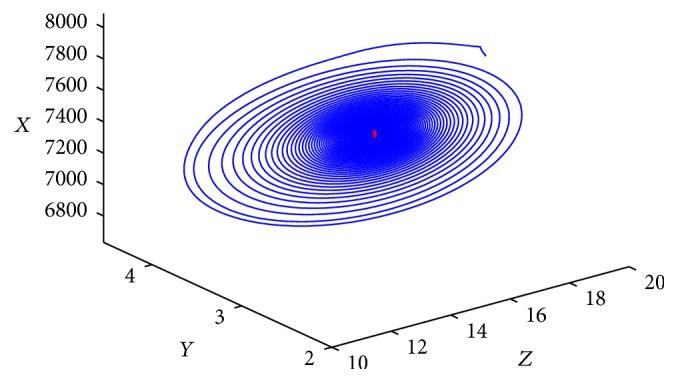
Phase trajectories of system (2) with *R*_1_ > 1, *τ* = 25 < *τ*_0_. The initial condition is (7970, 1000, 2.6, 0.2, 17).

**Figure 5:**
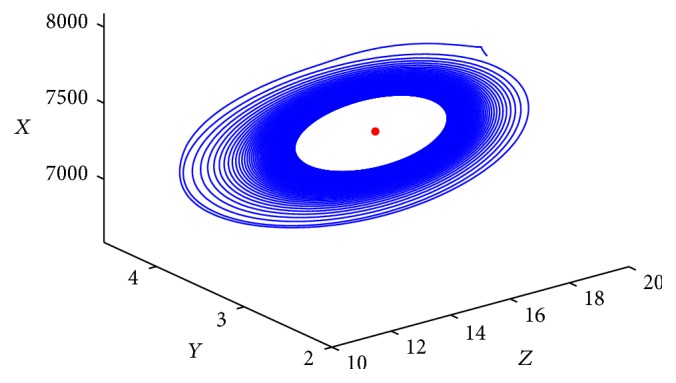
Phase trajectories of system (2) with *R*_1_ > 1, *τ* = 28 > *τ*_0_. The initial condition is (7970, 1000, 2.6, 0.2, 17).

Then we have that *R*_0_ = 3.2452 > 1, $\bar{R}=3.8942>1$, and *R*_1_ = 2.4119 > 1, (56) has two positive roots, and *h*′(*ν*_*k*_) ≠ 0. By simple computations, we have *ω*_0_ ≈ 0.0394 and *τ*_0_ ≈ 27.2546. From Theorem 9(ii), the infected equilibrium with CTL response *E* ^*∗*^ = (7363.6364,1180.0699,3.3333,16.6667,15.4021) is asymptotically stable if 0 < *τ* < *τ*_0_ and unstable if *τ* > *τ*_0_.  Figure 4 gives the phase trajectories of system (2) with *τ* < *τ*_0_ and suitable initial condition.  Figure 5 gives the phase trajectories of system (2) with *τ* > *τ*_0_ and suitable initial condition and shows the occurrence of the Hopf bifurcations.

**Figure 6:**
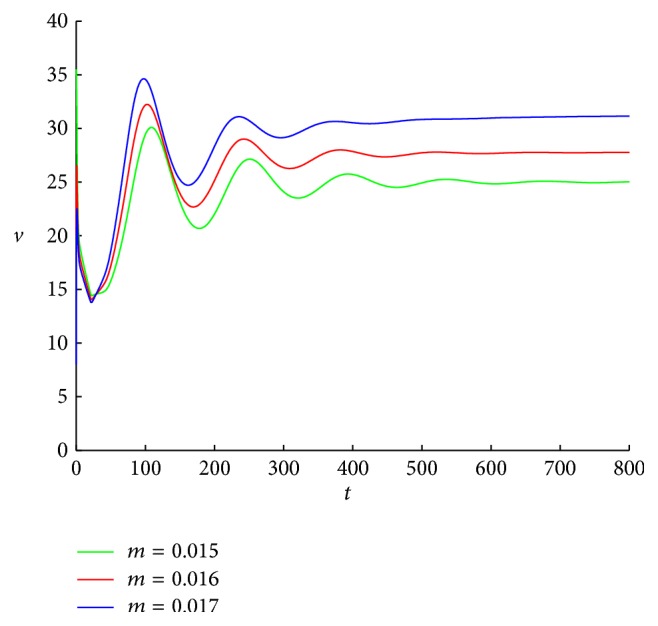
The curves of the free virus of system (2) with *τ* = 20, *m* = 0.015, 0.016, and 0.017. The initial conditions are chosen as (7000,1000,8, 35,10), (7000,1000,8, 20,10), and (7000,1000,8, 8,10), respectively.

**Figure 7:**
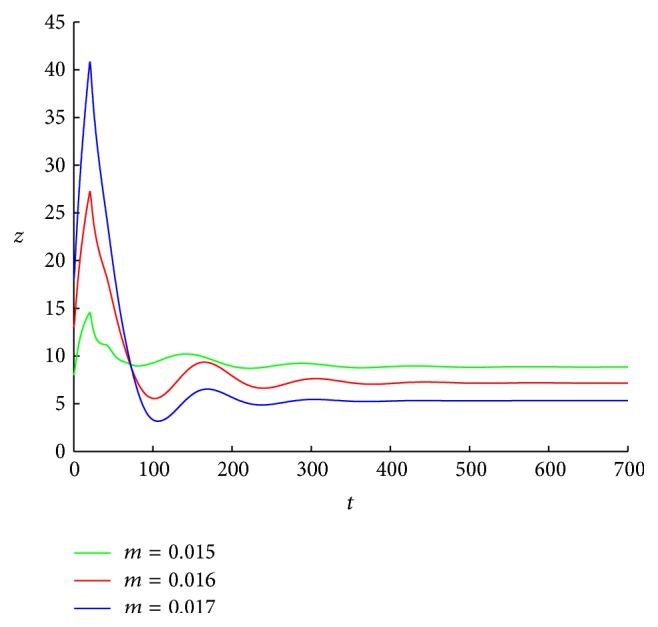
The curves of the CTLs of system (2) with *τ* = 20, *m* = 0.015, 0.016, and 0.017. The initial conditions are chosen as (7000,1000,8, 2,8), (7000,1000,8, 2,13), and (7000,1000,8, 2,18), respectively.

Since *v* ^*∗*^ = *kμ*_5_/(*μ*_4_(*q* − *m*)) and *z* ^*∗*^ = *μ*_1_ *μ*_3_ *μ*_4_(*q* − *m*)(*R*_1_ − 1)/(*pμ*_1_ *μ*_4_(*q* − *m*) + *pβkμ*_5_), it is easy to see that the number of free viruses is increased and the number of CTLs is decreased with respect to the immune impairment rate *m*. For example, if we choose *m* = 0.015, then *v*_1_^*∗*^ = 25 and *z*_1_^*∗*^ = 8.8462. If we choose *m* = 0.016, then *v*_2_^*∗*^ = 27.7778 and *z*_2_^*∗*^ = 7.1691. If we choose *m* = 0.017, then *v*_3_^*∗*^ = 31.25 and *z*_3_^*∗*^ = 5.3283.  Figure 6 shows that, for any initial conditions, when *m* = 0.015,0.016,0.017, the numbers of free viruses trend to *v*_1_^*∗*^, *v*_2_^*∗*^, and *v*_3_^*∗*^, respectively.  Figure 7 shows that, for any initial conditions, when *m* = 0.015,0.016,0.017, the numbers of CTLs trend to *z*_1_^*∗*^, *z*_2_^*∗*^, and *z*_3_^*∗*^, respectively. In Figures 6 and 7, all the data are chosen as in Figure 4 except the immune impairment rate *m*.

As immune impairment rate *m* increases, the CTL response gradually becomes weak and the individuals eventually develop AIDS. Thus, in order to control the HIV infection, we should decrease the value of *m*. Numerical simulations show the similar known results (see, e.g., [22]).
